# Development of a weighted Alpha-Fetoprotein tumor burden score-integrated nomogram for predicting overall survival in locally ablated hepatocellular carcinoma patients

**DOI:** 10.3389/fonc.2025.1660569

**Published:** 2025-10-06

**Authors:** Yang Wang, Zhixia Gu, Wenying Qiao, Xiaoxue Yuan, Caixia Hu, Ronghua Jin

**Affiliations:** ^1^ National Key Laboratory of Intelligent Tracking and Forecasting for Infectious Diseases, Beijing Ditan Hospital, Capital Medical University, Beijing, China; ^2^ Beijing Institute of Infectious Diseases, Beijing, China; ^3^ National Center for Infectious Diseases, Beijing Ditan Hospital, Capital Medical University, Beijing, China; ^4^ Beijing Key Laboratory of Emerging Infectious Diseases, Institute of Infectious Diseases, Beijing Ditan Hospital, Capital Medical University, Beijing, China; ^5^ Interventional Therapy Center for Oncology, Beijing You’an Hospital, Capital Medical University, Beijing, China

**Keywords:** hepatocellular carcinoma, Weighted Alpha-Fetoprotein Tumor Burden Score, overall survival, nomogram, local ablation

## Abstract

**Introduction:**

The Weighted Alpha-Fetoprotein Tumor Burden Score (WATS) shows promise for hepatocellular carcinoma (HCC) prognosis, but its usefulness in local ablation patients is uncertain, and no validated nomograms exist for overall survival (OS) prediction.

**Methods:**

This retrospective study enrolled 862 HCC patients who underwent local ablation therapy at Beijing You’an Hospital between January 1, 2015 and December 31, 2022. Participants were randomly allocated into a training cohort (n=603) and validation cohort (n=259) in a 7:3 ratio. Based on the median value of the WATS score, patients were stratified into low-risk (n=431) and high-risk (n=431) groups. The Kaplan-Meier (KM) curve was used to compare the prognosis between the two groups. Potential prognostic factors were screened via least absolute shrinkage and selection operator (Lasso) regression, followed by construction of a WATS-incorporated nomogram prediction model using Cox proportional hazards regression. The SHapley Additive exPlanations (SHAP) method was employed to interpret variable contributions within the model. Model performance was evaluated via Receiver operating characteristic (ROC) curve, calibration curve, and decision curve analysis (DCA). Patients were stratified into low- and high-risk groups according to the nomogram scores, and KM curves were used to compare OS differences between the two groups.

**Results:**

The study identified the WATS, age, history of drinking, and prealbumin as independent prognostic factors for OS, and successfully established a nomogram model for OS prediction. The ROC curves, calibration curves, and DCA all confirmed that the model possesses good discriminative ability, calibration accuracy, and clinical utility. KM curves demonstrated that the nomogram could effectively stratify patients into different risk categories with satisfactory predictive performance.

**Conclusion:**

This study developed and validated a novel prognostic nomogram incorporating the WATS to assess OS in HCC patients receiving local ablation therapy. The nomogram demonstrated robust discriminative ability, enabling accurate prediction of 3-, 5-, and 8-year OS rates, thereby providing clinicians with a reliable tool for individualized risk assessment and treatment decision-making.

## 1 Introduction

Hepatocellular carcinoma (HCC) represents over 90% of primary liver cancers and remains a major global health concern (1). Globally, it is the sixth most frequently diagnosed malignancy and the third leading cause of cancer mortality (2). For early-stage HCC, local ablation serves as a first-line treatment, and recent advancements in this technique have contributed to improved patient survival (3). This minimally invasive approach offers benefits such as reduced surgical trauma, minimal bleeding, and fewer complications (4). Despite these advantages, post-ablation outcomes remain suboptimal, with a 5-year overall survival (OS) rate of only 30-40% (5, 6). Given the widespread prevalence and unfavorable outcomes of HCC patients who received local ablation, developing prognostic models to predict OS is essential.

Accurate and objective assessment of tumor burden has long been a major challenge in oncology. In 2009, Mazzaferro et al. conceptualized tumor burden through the Metroticket model, framing tumor progression as a journey in which higher tumor burden corresponds to a greater cost to survival (7). Building on this concept, Sasaki et al. developed the Tumor Burden Score (TBS), a composite index that integrates tumor diameter and number using the Pythagorean theorem (8). Subsequent studies, including work by Tsilimigras et al., demonstrated the utility of TBS in stratifying prognosis among patients undergoing resection for HCC, effectively reducing heterogeneity within early- and intermediate-stage disease (9).

While TBS provides a simple and practical anatomical assessment of tumor burden, it does not fully account for biological aggressiveness—such as alpha-fetoprotein (AFP) levels, tumor differentiation, or microvascular invasion. To address this limitation, the Weighted Alpha-fetoprotein Tumor burden Score (WATS) was introduced. Originally developed by Lu et al. in a cohort of 772 HCC patients undergoing curative hepatectomy, WATS integrates both tumor burden and biological invasiveness into a single continuous index (10). Through multivariable Cox regression, three independent predictors of progression-free survival—tumor number, tumor size, and the natural logarithm of AFP (ln AFP)—were identified and assigned statistically derived weights of 0.73, 0.17, and 0.10, respectively, normalized to sum to 1. The resulting formula, WATS = 0.73 × tumor number + 0.17 × tumor size + 0.10 × ln AFP, generates a weighted prognostic score that reflects both anatomical extent and molecular characteristics, with higher values indicating greater disease aggressiveness (10). Unlike conventional staging systems, such as Tumor-Node-Metastasis (TNM) or Barcelona Clinic Liver Cancer (BCLC), which rely on categorical definitions and arbitrary thresholds, WATS leverages continuous variables and data-driven weighting to enable more granular and biologically informed risk stratification. By simultaneously capturing tumor morphology and serological biomarkers within a unified framework, WATS overcomes key limitations of earlier models such as TBS (11, 12). Validated in both training and internal cohorts, WATS demonstrates improved prognostic resolution, enhances the accuracy of survival prediction, and supports more individualized clinical decision-making—particularly in complex cases where traditional systems may lack discriminatory power (10).

Nevertheless, as for HCC patients treated with local ablation, the predictive capability of WATS for prognosis remains uncertain. Additionally, there is also lacking effective nomograms to predict patients’ mortality rates. Therefore, this study included WATS in the analysis and investigated its association with OS. Subsequently, a nomogram for HCC patients after local ablation was built based on clinical variables selected by Least Absolute Shrinkage and Selection Operator (Lasso) Cox regression. Moreover, the comparison of survival times among different risk groups derived from the established nomogram was done, aiming to facilitate the identification of high-risk populations and offer more precise clinical guidance.

## 2 Methods

### 2.1 Study populations

This study enrolled 862 HCC patients who received ablation treatment at Beijing You’an Hospital, Capital Medical University, between January 1, 2015, and December 31, 2022. The diagnosis of HCC was according to the American Association for the Study of Liver Disease (AASLD) guideline. The inclusion criteria were as follows: (1) age from 18 to 80 years; (2) complete clinical and follow-up data; (3) BCLC stage 0, A or B; (4) Child-Pugh class A or B. Exclusion criteria: Patients were excluded if they met any one of the following criteria: (1) received other treatment before ablation; (2) distant metastasis of HCC; (3) secondary liver cancer; (4) coagulation function disorders or serious diseases of vital organs, such as the heart, brain, lung, and kidney.

Approval for this research was obtained from the Ethics Committee of Beijing You’an Hospital, Capital Medical University, ensuring adherence to the Declaration of Helsinki guidelines. The requirement for informed consent was waived due to the retrospective nature of this study.

### 2.2 Clinical characteristics and follow-up

Clinical characteristics were gathered before the local ablation, including: (1) demographic data: age, gender, history of smoking, drinking, antiviral therapy, hypertension, and diabetes; (2) tumor information: tumor number (TN), tumor size (TS) and AFP. (3) liver function indicators: Child-Pugh class, history of liver cirrhosis, alanine aminotransferase (ALT), aspartate transaminase (AST), total bilirubin (TBIL), direct bilirubin (DBIL), gamma-glutamyl transferase (GGT), albumin (ALB), alkaline phosphatase (ALP), prealbumin (Palb) and globulin (Glob). (4) other characteristics: prothrombin time (PT), thrombin time (TT), prothrombin time activity (PTA), activated partial thromboplastin time (APTT), red blood cell (RBC), white blood cell (WBC), and hemoglobin (Hb).

Tumor number and maximum tumor diameter were assessed based on preoperative multimodal imaging, including contrast-enhanced ultrasound, computed tomography (CT), and/or magnetic resonance imaging (MRI). These evaluations were performed by experienced radiologists who were blinded to the patients’ pathological outcomes and clinical prognoses, thereby minimizing interpretation bias and ensuring assessment consistency. Preoperative blood samples were collected and analyzed at our institutional central laboratory using standardized platforms and commercial assay kits to measure AFP, liver function parameters, and coagulation profiles. This centralized testing approach was implemented to reduce inter-assay variability and ensure data uniformity and reliability across all study participants.

In our study, the previously reported WATS score was used as the core variable to stratify patients into high-risk and low-risk groups according to its median value, for the prediction of OS in HCC patients undergoing local ablation. WATS = 0.73 × tumor number + 0.17 × tumor size + 0.10 × ln AFP (10).

Patients with HCC were recommended to undergo regular follow-ups in line with clinical guidelines after local ablation. Typically, these patients were followed up 3 to 6 months, which comprised clinical characteristics and adverse events. OS, as the primary endpoint of this study, was defined as the interval from local ablation to either the occurrence of death or the last follow-up.

### 2.3 Ablation procedure

All enrolled patients were treated with local ablation, which was performed by qualified hepatologists and interventional radiologists. The specific process includes 5 items: (1) Appropriate position for ablation was determined by CT or MRI. (2) The ablation needle was inserted in the marked skin, and followed by image scanning to track the ablation process. (3) For the purpose of attaining complete ablation, operators should expand the ablative range and contemplate multiple sites, overlapping, or repeated ablation. (4) In order to prevent tumor implantation and postoperative bleeding, the needle track required to be heated in the final stage. (5) Following the ablation, all patients underwent imaging examinations to assess treatment efficacy and possible complications.

### 2.4 Statistical analysis

Categorical variables were expressed as frequencies (percentages), whereas continuous variables were reported as means ± standard deviation or medians (quartiles). Comparisons between two groups were analyzed by Student’s t test, Chi-square test, or non-parametric test as appropriate. All statistical analyses were conducted using R software (version 4.5.0). All tests were two-tailed and statistical significance was set at *P* < 0.05.

Firstly, the Kaplan-Meier (KM) curve and log-rank test were used to assess the performance of WATS score in HCC OS. To address potential multicollinearity among variables, select the most predictive and parsimonious set of features, and mitigate the risk of overfitting, we employed Lasso regression analysis, incorporating 10-fold cross-validation. The optimal value of the regularization parameter (λ) was determined by minimizing the cross-validated partial likelihood deviance. Variables retaining non-zero coefficients after Lasso penalization were selected as candidate predictors. These Lasso-selected variables were subsequently incorporated into a multivariate Cox proportional hazards regression model. Variables that remained statistically significant (typically defined as *P* < 0.05) in this multivariate model were identified as independent prognostic factors for OS. Based on the results of the final multivariate Cox regression model, we constructed a nomogram using the rms package in R software. This nomogram provides a graphical representation of the multivariate Cox model. Each independent prognostic factor, including the WATS score as a key variable, is assigned a specific point value on the “Points” scale at the top of the nomogram. The points for each predictor are summed to yield a total point score. This total score is then projected downward to the lower scales of the nomogram to estimate the probability of OS at specific time points (3-year, 5-year, and 8-year). In order to determine the relative importance of the variables, we employed a statistical machine learning approach, Shapley Additive Explanations (SHAP). Furthermore, receiver operating characteristic curves (ROC) were plotted and the area under the curves (AUC) were calculated to evaluate the discrimination of the model. The calibration and decision curve analysis (DCA) were then used to validate the calibration performance and clinical utility. At last, patients were classified into low-risk and high-risk groups on the basis of the nomogram. KM curves were applied to compare the OS between two groups.

## 3 Results

### 3.1 Baseline characteristics

During the period from January 1, 2015, to December 31, 2022, a total of 862 HCC patients after local ablation were recruited in this study and randomized into two groups with a 7:3 ratio. There were 603 patients constituted the training set, while 259 formed the validation set (**Table 1**). The result revealed no statistical differences between the two groups, except the history of diabetes. Our research concluded patients’ last follow-up on July 1, 2024, with a median follow-up duration of 52.7 months.

**Table 1:** Clinical characteristics for training and validation cohorts.

Characteristic	Training cohort (N = 603)	Validation cohort (N = 259)	*P* value
Age	56.78 ± 8.99	56.17 ± 9.13	0.363
Gender (%)			0.808
Male	481 (79.8)	204 (78.8)	
Female	122 (20.2)	55 (21.2)	
Hypertension (%)			0.415
No	453 (75.1)	187 (72.2)	
Yes	150 (24.9)	72 (27.8)	
Diabetes (%)			**0.034**
No	465 (77.1)	217 (83.8)	
Yes	138 (22.9)	42 (16.2)	
Antiviral (%)			0.667
No	279 (46.3)	115 (44.4)	
Yes	324 (53.7)	144 (55.6)	
Smoking (%)			0.854
No	341 (56.6)	144 (55.6)	
Yes	262 (43.4)	115 (44.4)	
Drinking (%)			0.128
No	411 (68.2)	162 (62.5)	
Yes	192 (31.8)	97 (37.5)	
Cirrhosis (%)			0.924
No	83 (13.8)	37 (14.3)	
Yes	520 (86.2)	222 (85.7)	
Child-Pugh (%)			0.821
A	460 (76.3)	195 (75.3)	
B	143 (23.7)	64 (24.7)	
AFP (ng/mL)	10.23 (3.70, 72.77)	12.17 (3.84, 54.54)	0.839
WBC (10^9/L)	5.25 ± 2.25	5.07 ± 2.07	0.274
RBC (10^12/L)	4.16 ± 0.62	4.19 ± 0.62	0.505
Hb (g/L)	129.91 ± 19.10	132.12 ± 18.91	0.119
ALT (U/L)	31.93 ± 20.88	32.74 ± 19.54	0.593
AST (U/L)	32.54 ± 16.29	32.26 ± 12.02	0.808
ALP (U/L)	87.09 ± 33.25	84.98 ± 39.89	0.422
GGT (U/L)	51.30 (32.25, 83.35)	50.70 (31.95, 85.65)	0.968
TBIL (umol/L)	18.84 ± 9.70	19.73 ± 10.27	0.224
DBIL (umol/L)	6.07 ± 4.25	6.68 ± 4.82	0.062
ALB (g/L)	37.28 ± 4.63	37.47 ± 4.80	0.588
Glob (g/L)	28.34 ± 5.60	27.65 ± 5.02	0.091
APTT (s)	33.76 ± 4.69	33.48 ± 4.48	0.421
PT (s)	12.45 ± 1.41	12.41 ± 1.59	0.674
TT (s)	15.88 ± 2.36	15.90 ± 2.27	0.906
WATS	1.82 ± 0.74	1.78 ± 0.71	0.521

AFP, Alpha-Fetoprotein; WBC, white blood cell; RBC, red blood cell; Hb, Hemoglobin; ALT, alanine aminotransferase; AST, aspartate transferase; ALP, alkaline phosphatase; GGT, gamma-glutamyl transferase; TBIL, total bilirubin; DBIL, direct bilirubin; ALB, albumin; Glob, globulin; APTT, activated partial thromboplastin time; PT, prothrombin time; TT, thrombin time; WATS, Weighted Alpha-Fetoprotein Tumor Burden Score.

Bold values indicate statistically significant results with *P* < 0.05.

Among these patients, the average age was 56.60 years, with 685 (79.5%) males and 177 (20.5%) females. Notably, 377 (43.7%) patients had a history of smoking and 289 (33.5%) had a history of drinking. Furthermore, 222 (25.8%) individuals were diagnosed with hypertension and 180 (20.9%) with diabetes mellitus. The training and validation cohorts both had a majority of patients in Child-Pugh class A (76.3% and 75.3%, respectively).

### 3.2 The performance of WATS for predicting the OS

According to the median value of WATS score, patients could be classified into low-risk group (n=431) and high-risk group (n=431). The comparison of baseline characteristics between two groups was shown in **Table 2**. There were significant differences between two groups in terms of the WATS score.

**Table 2:** Patient characteristics between low-risk group and high-risk group.

Characteristic	All patients (n=862)	Low-risk patients (n=431)	High-risk patients (n=431)	*P* value
Age	56.60 ± 9.03	56.37 ± 9.09	56.83 ± 8.97	0.451
Gender (%)				0.129
Male	685 (79.5)	333 (77.3)	352 (81.7)	
Female	177 (20.5)	98 (22.7)	79 (18.3)	
Hypertension (%)				0.938
No	640 (74.2)	319 (74.0)	321 (74.5)	
Yes	222 (25.8)	112 (26.0)	110 (25.5)	
Diabetes (%)				0.154
No	682 (79.1)	332 (77.0)	350 (81.2)	
Yes	180 (20.9)	99 (23.0)	81 (18.8)	
Antiviral (%)				**0.001**
No	394 (45.7)	172 (39.9)	222 (51.5)	
Yes	468 (54.3)	259 (60.1)	209 (48.5)	
Smoking (%)				0.099
No	485 (56.3)	255 (59.2)	230 (53.4)	
Yes	377 (43.7)	176 (40.8)	201 (46.6)	
Drinking (%)				0.083
No	573 (66.5)	299 (69.4)	274 (63.6)	
Yes	289 (33.5)	132 (30.6)	157 (36.4)	
Cirrhosis (%)				0.922
No	120 (13.9)	61 (14.2)	59 (13.7)	
Yes	742 (86.1)	370 (85.8)	372 (86.3)	
Child-Pugh (%)				0.873
A	655 (76.0)	329 (76.3)	326 (75.6)	
B	207 (24.0)	102 (23.7)	105 (24.4)	
AFP (ng/mL)	10.70 (3.71, 65.34)	5.12 (2.90, 16.35)	32.86 (6.82, 294.65)	**<0.001**
WBC (10^9/L)	5.20 ± 2.20	5.12 ± 2.22	5.28 ± 2.18	0.275
RBC (10^12/L)	4.16 ± 0.62	4.17 ± 0.63	4.16 ± 0.60	0.954
Hb (g/L)	130.57 ± 19.06	131.01 ± 19.42	130.14 ± 18.70	0.503
ALT (U/L)	32.17 ± 20.48	29.83 ± 18.23	34.52 ± 22.28	**0.001**
AST (U/L)	32.45 ± 15.64	31.10 ± 14.58	33.81 ± 16.53	**0.011**
ALP (U/L)	86.45 ± 35.36	83.91 ± 32.57	89.00 ± 37.81	**0.034**
GGT (U/L)	51.20 (32.05, 83.95)	46.20 (28.90, 78.85)	58.00 (35.05, 90.85)	**<0.001**
TBIL (μmol/L)	19.11 ± 9.88	19.56 ± 10.03	18.66 ± 9.71	0.181
DBIL (μmol/L)	6.25 ± 4.43	6.52 ± 4.67	5.99 ± 4.17	0.081
ALB (g/L)	37.33 ± 4.68	37.72 ± 4.73	36.95 ± 4.60	**0.015**
Glob (g/L)	28.13 ± 5.44	28.21 ± 5.25	28.05 ± 5.63	0.683
APTT (s)	33.68 ± 4.62	33.62 ± 4.47	33.74 ± 4.78	0.693
PT (s)	12.44 ± 1.47	12.45 ± 1.59	12.43 ± 1.34	0.898
TT (s)	15.88 ± 2.33	15.93 ± 2.22	15.84 ± 2.44	0.584

AFP, Alpha-Fetoprotein; WBC, white blood cell; RBC, red blood cell; Hb, Hemoglobin; ALT, alanine aminotransferase; AST, aspartate transferase; ALP, alkaline phosphatase; GGT, gamma-glutamyl transferase; TBIL, total bilirubin; DBIL, direct bilirubin; ALB, albumin; Glob, globulin; APTT, activated partial thromboplastin time; PT, prothrombin time; TT, thrombin time.

Bold values indicate statistically significant results with *P* < 0.05.

**Figure 1:**
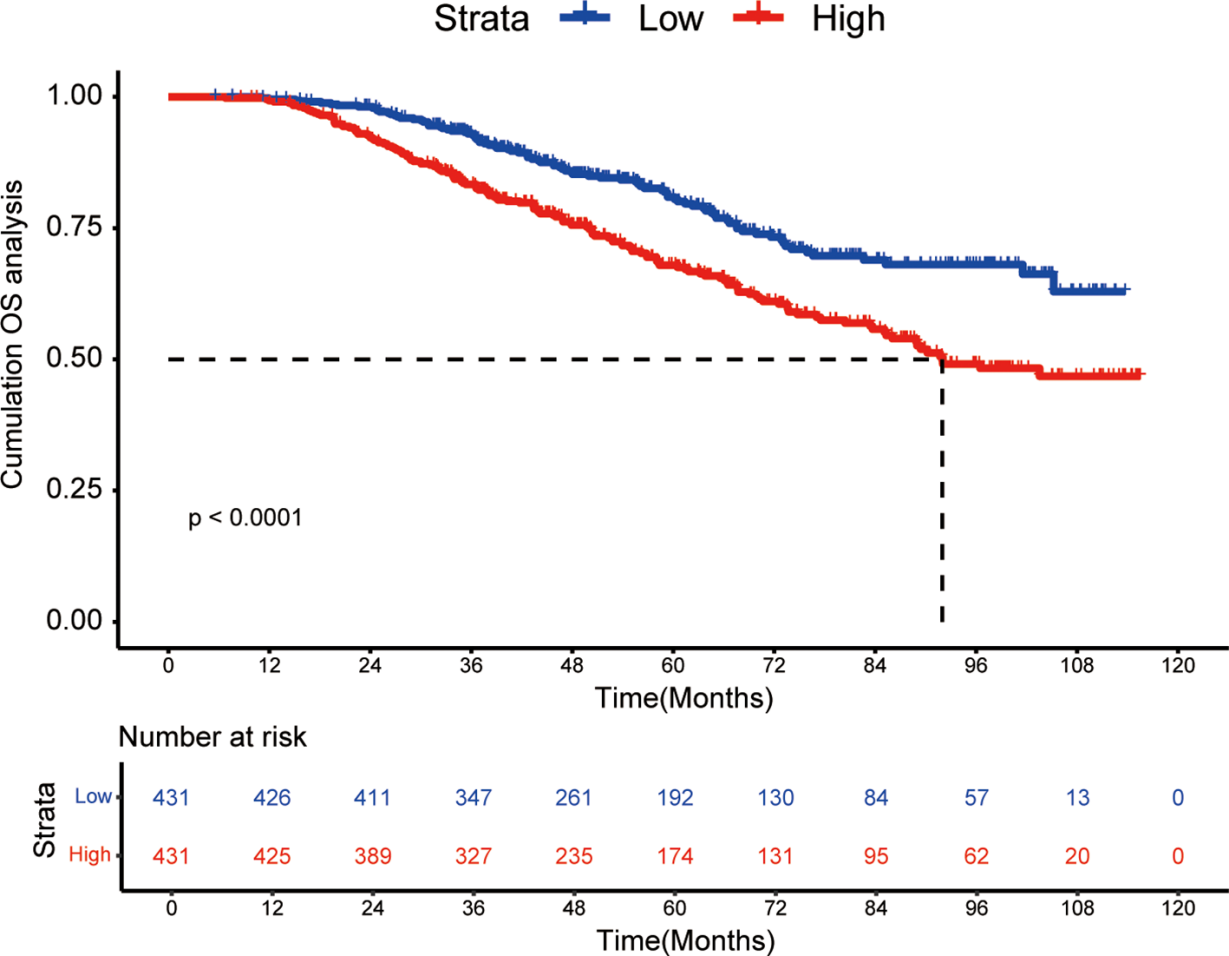
Kaplan-Meier curves of high WATS group and low WATS group. WATS: Weighted Alpha-Fetoprotein Tumor Burden Score; OS, overall survival.

The KM curve was plotted in **Figure 1** for comparing the prognosis between two groups. The median OS was not reached in low-risk group and 92.0 months (95% CI: 85.1 months to not reached) in high-risk group, respectively. The cumulative OS rates for 3-, 5-, 8-year were 93.0%, 80.9% and 68.1% in the low-risk group, while 83.3%, 67.9% and 49.1% in the high-risk group (*P* < 0.0001).

### 3.3 Independent prognostic factors associated with OS

**Figure 2:**
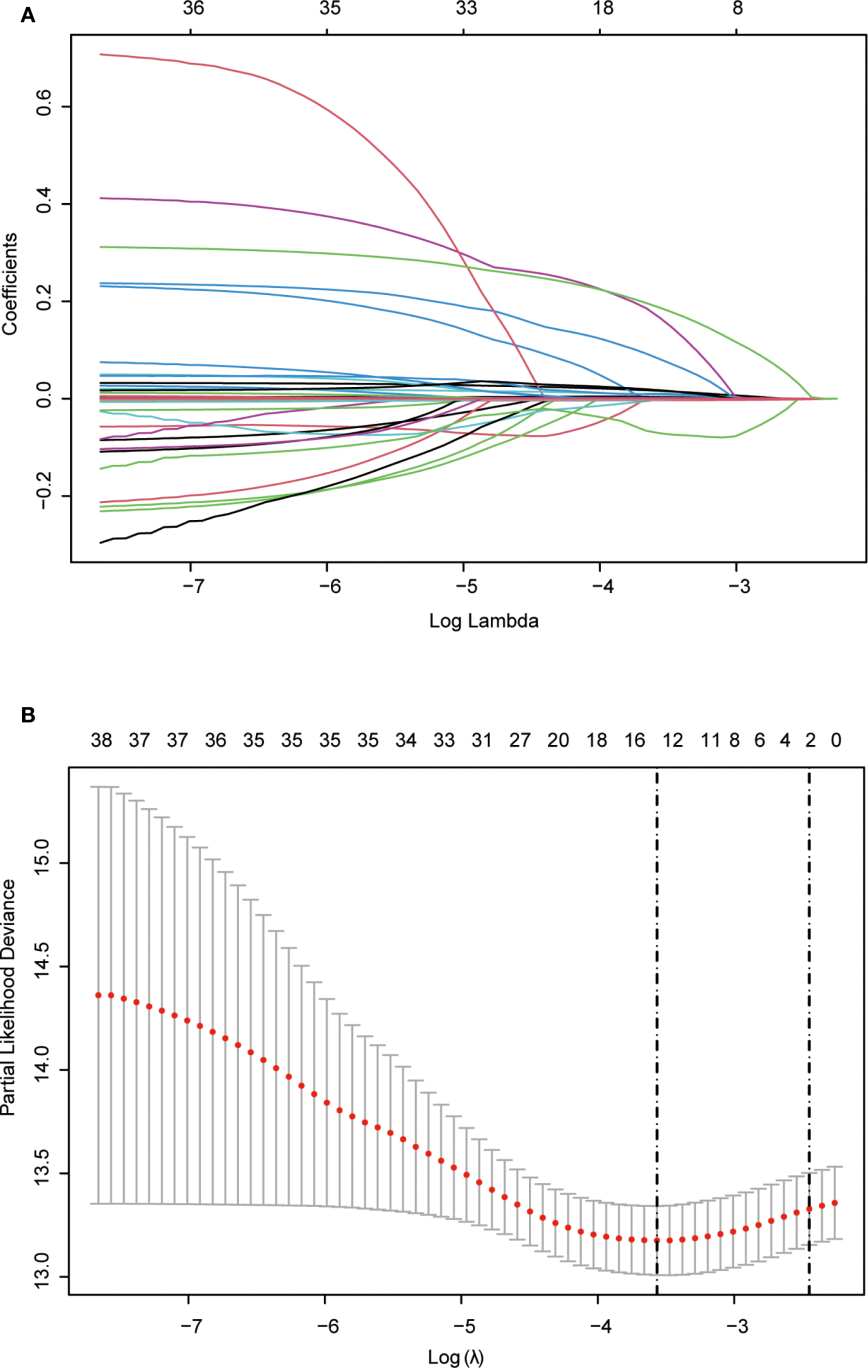
Lasso regression employed to further identified risk factors for OS. **(A)** Lasso regression coefficients paths; **(B)** Lasso regression cross-validation curve. Lasso: least absolute shrinkage and selection operator; OS, overall survival.

Lasso regression, utilizing a loss function with L1 regularization to penalize model coefficients while minimizing the objective function, was employed to screen risk factors associated with OS (**Figure 2**). The 10-fold cross-validation method was applied to select the optimal λ value, which was determined to be 0.0283 (Log λ = -1.548). Significant risk factors filtered by Lasso regression were age, history of drinking, Child-Pugh class, WBC, RBC, AST, Glob, GGT, Palb, PT, PTA, TT, and WATS. These variables were further incorporated into the multivariable Cox regression analysis, revealing that WATS (HR:1.334, 95% *CI*: 1.113-1.600), age (HR:1.031, 95% *CI*: 1.011-1.052), history of drinking (HR:1.449, 95% *CI*: 1.058-1.985) and Palb (HR:0.996, 95% *CI*: 0.992-0.999) as the independent prognostic factors for OS (**Table 3**).

**Table 3:** Cox proportional hazards regression to predict OS based on Lasso regression.

Variables	HR (95%CI)	*P* value
Age	1.031 (1.011-1.052)	**0.002**
Child-Pugh	1.219 (0.837-1.775)	0.301
Drinking	1.449 (1.058-1.985)	**0.021**
WBC	0.961 (0.889-1.038)	0.312
RBC	0.980 (0.713-1.347)	0.901
AST	1.007 (0.998-1.016)	0.113
GGT	1.002 (0.999-1.004)	0.076
Glob	1.016 (0.090-1.042)	0.236
Palb	0.996 (0.992-0.999)	**0.039**
PT	1.002 (0.715-1.403)	0.991
PTA	0.994 (0.962-1.028)	0.739
TT	1.038 (0.970-1.112)	0.277
WATS	1.334 (1.113-1.600)	**0.002**

WBC, white blood cell; RBC, red blood cell; AST, aspartate transferase; GGT, gamma-glutamyl transferase; Glob, globulin; Palb, prealbumin; PT, prothrombin time; PTA, prothrombin time activity; TT, thrombin time; WATS, Weighted Alpha-Fetoprotein Tumor Burden Score; Lasso, least absolute shrinkage and selection operator; OS, overall survival.

Bold values indicate statistically significant results with *P* < 0.05.

**Figure 3:**
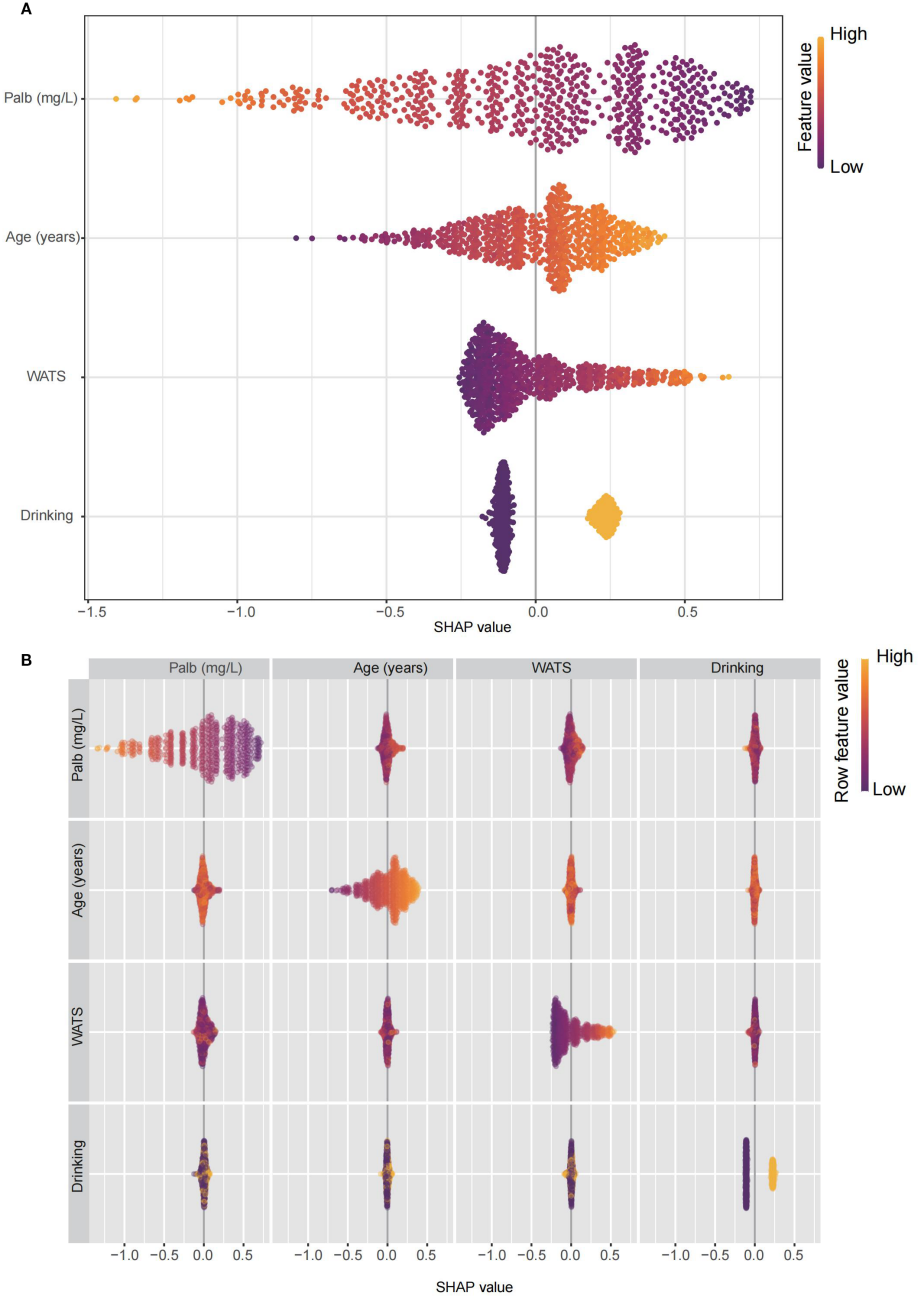
The constructed SHAP plot quantified the contribution of each feature to the model. **(A)** The SHAP plot illustrated the key risk factors identified through the Lasso-Cox model. **(B)** The correlation heatmap displayed the correlations among these factors. Lasso: least absolute shrinkage and selection operator; SHAP, SHapley Additive exPlanations.

In order to assess the specific impact of each variable as a risk factor on OS, we employed the global interpretation method to construct SHAP. This approach quantifies the contribution of each feature to the model and identifies the prediction contribution of the model to a broader range of outcomes. The risk factors in Lasso-cox were further presented (**Figure 3A**), and the correlation heat map shows the correlation between factors (**Figure 3B**).

### 3.4 Development of the nomogram

**Figure 4:**
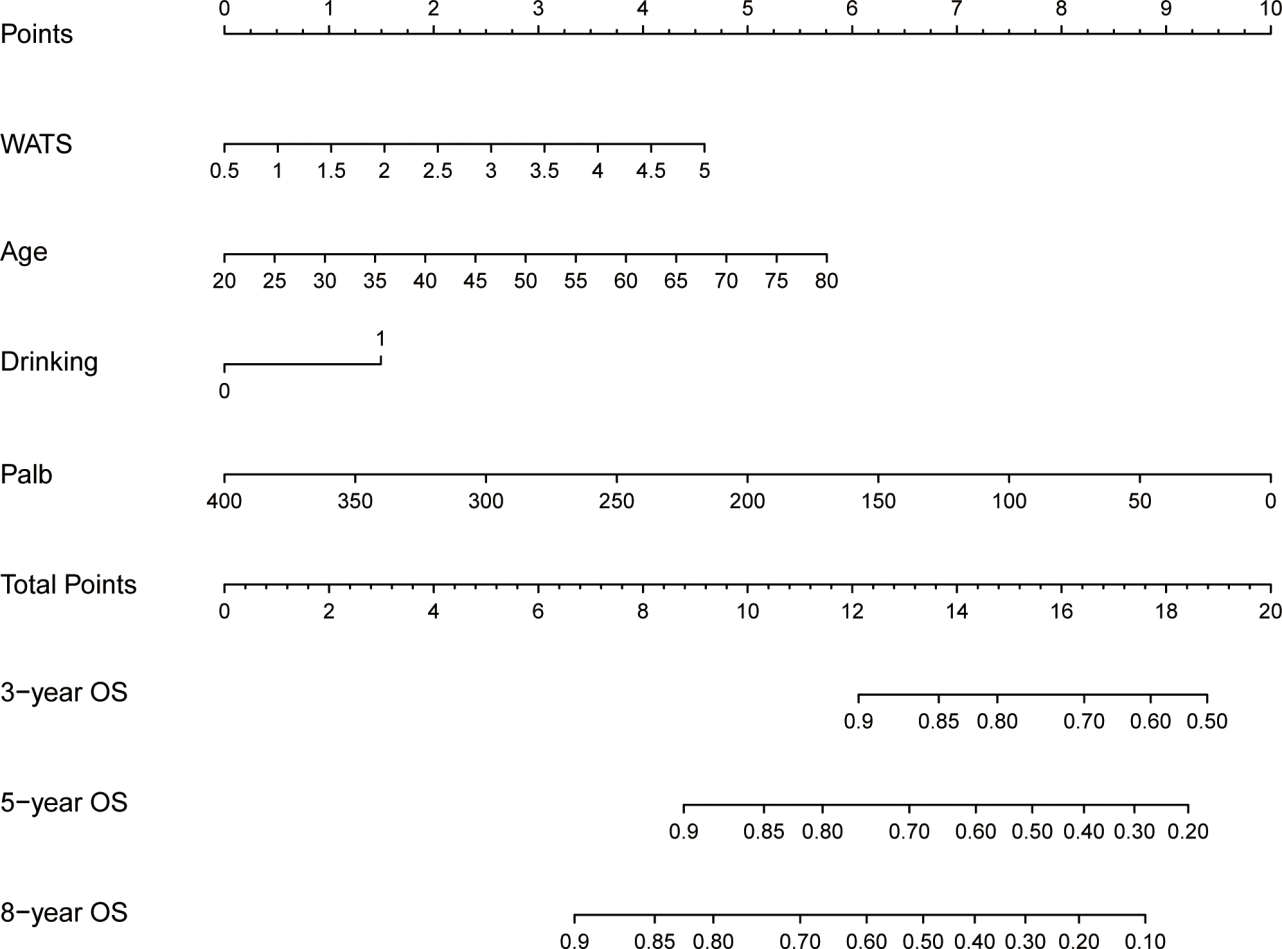
Nomogram for predicting 3-, 5-, and 8-year OS in locally ablated hepatocellular carcinoma patients. OS, overall survival; Palb, prealbumin; WATS, Weighted Alpha-Fetoprotein Tumor Burden Score.

Based on these independent prognostic factors, we constructed a nomogram for predicting the OS of HCC patients who received local ablation (**Figure 4**). Every risk factor corresponds to a specific score according to its value on the nomogram. It was necessary to sum the scores of factors and draw a vertical line at the corresponding total point. After these steps, the vertical line intersects with three lines representing mortality risk, which forecast the 3-, 5-, and 8-year OS.

**Figure 5:**
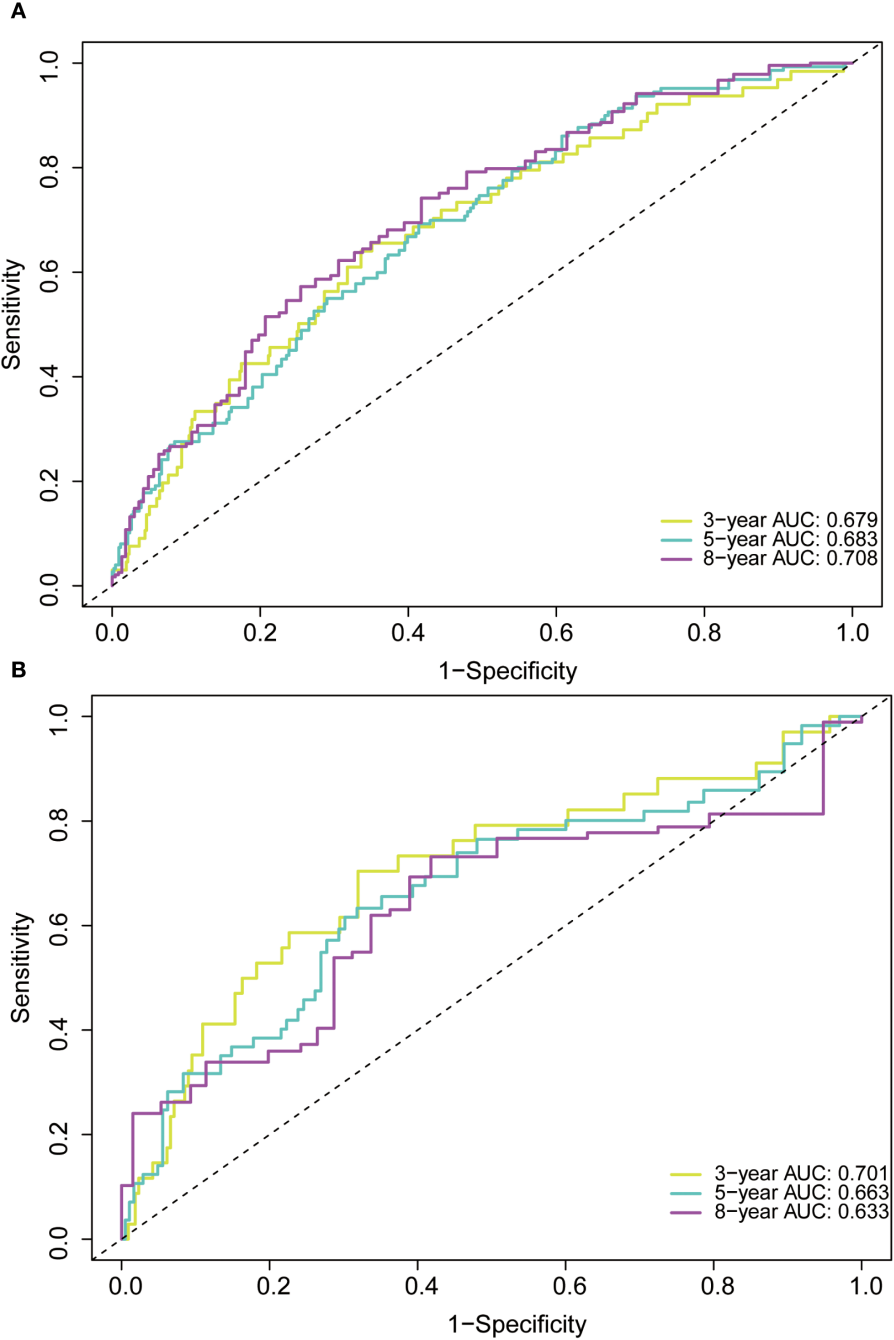
Receiver operating characteristic (ROC) curve of the nomogram in the training **(A)** and validation **(B)** cohorts. AUC, area under the curve.

**Figure 6:**
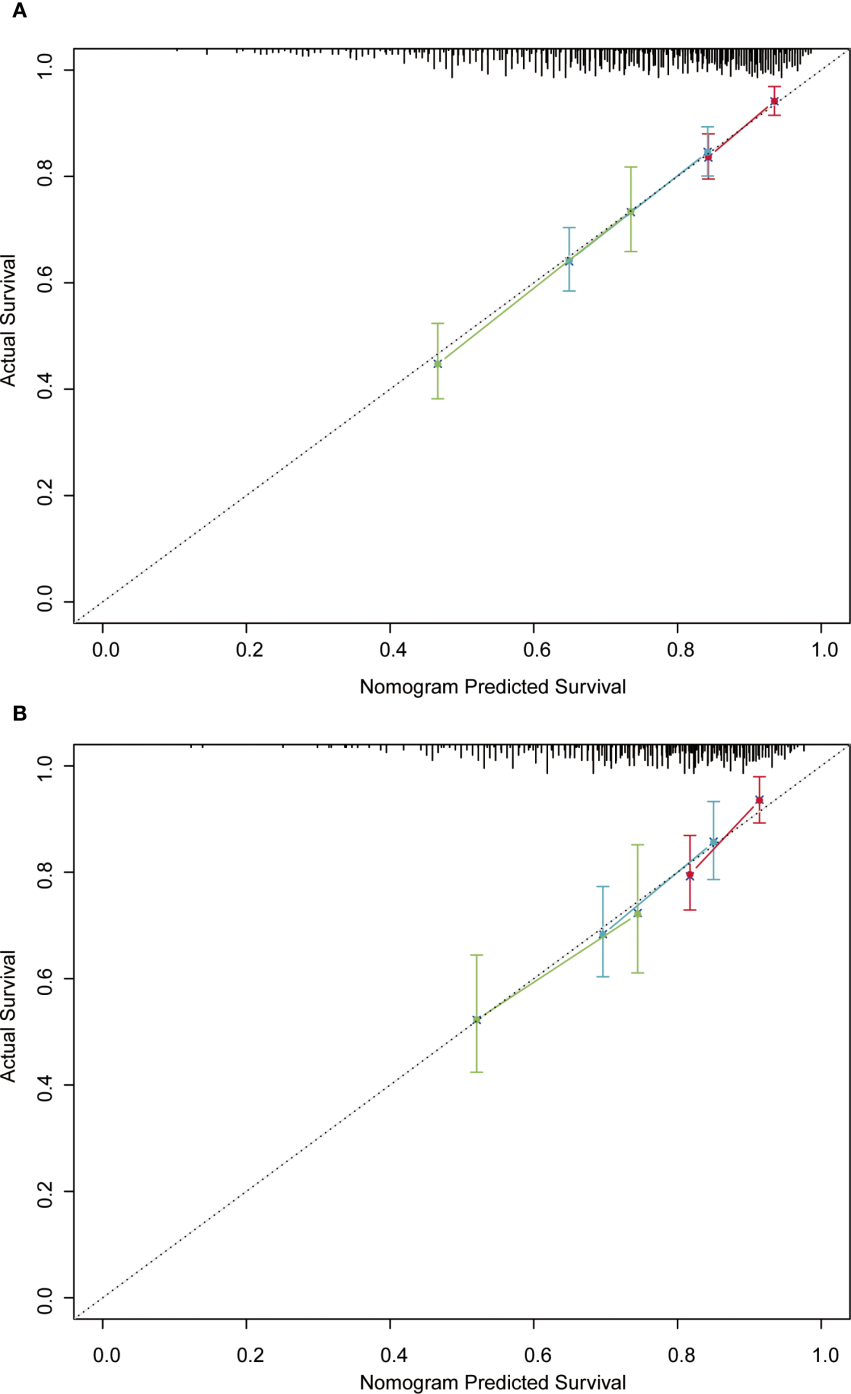
Calibration curves of the nomogram in the training **(A)** and validation **(B)** cohorts.

**Figure 7:**
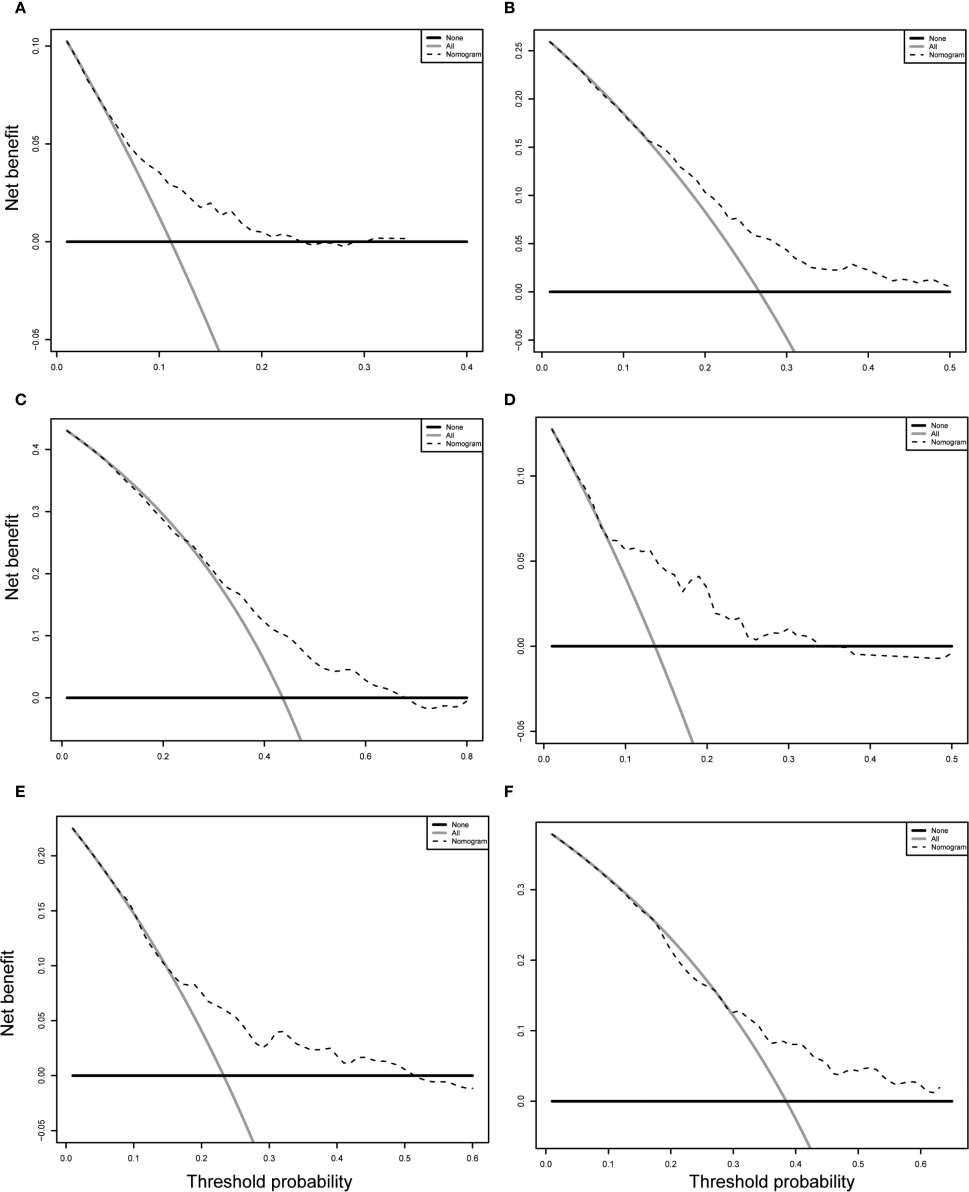
Decision curve analysis (DCA) of the nomogram in the training **(A-C)** and validation **(D-F)** cohorts. **(A-C)** DCA curve for predicting 3-year OS **(A)**, 5-year OS **(B)** and 8-year OS **(C)** in training cohort. **(D-F)** DCA curve for predicting 3-year OS **(D)**, 5-year OS **(E)** and 8-year OS **(F)** in validation cohort. OS, overall survival.

**Figure 8:**
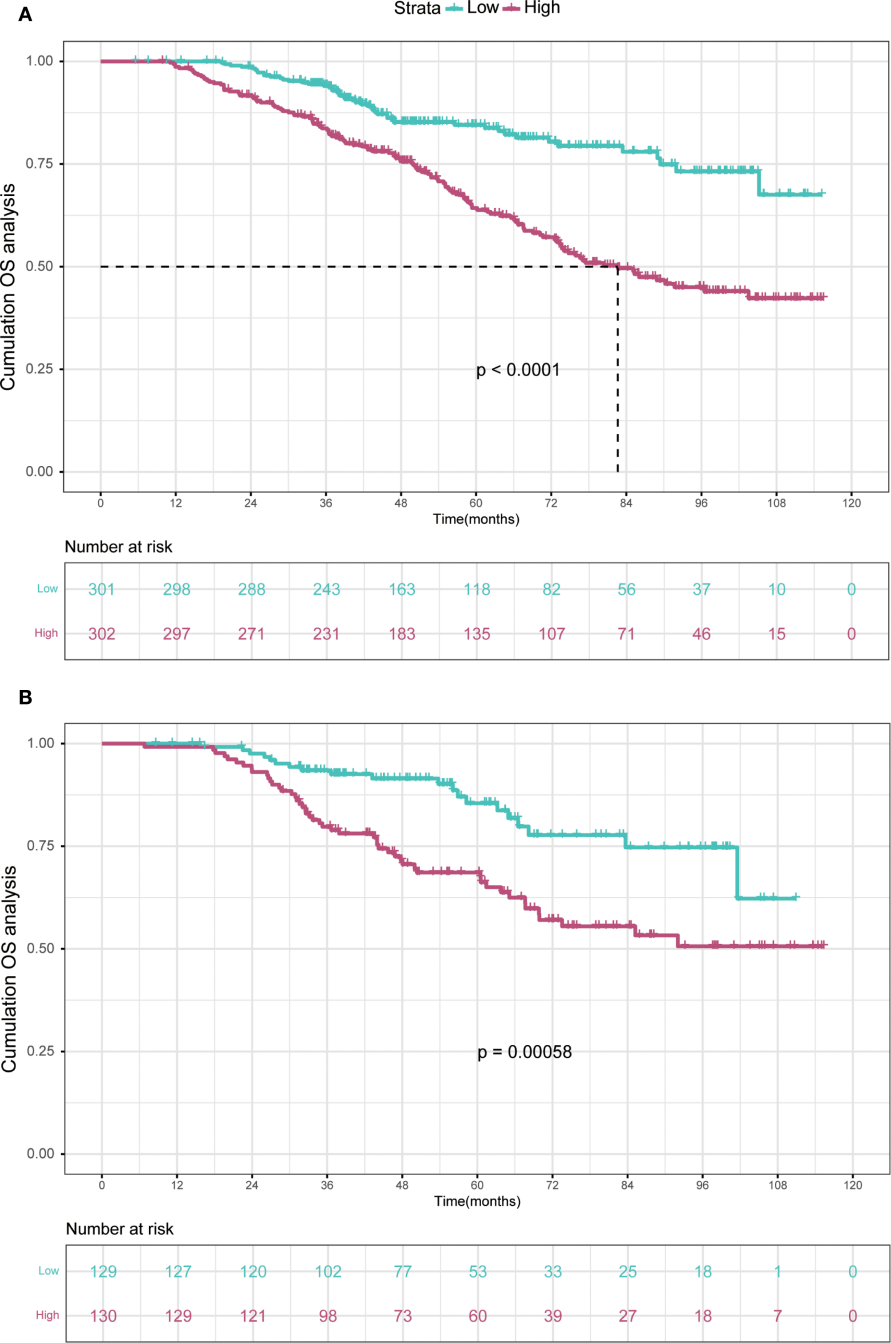
Kaplan-Meier curves depict OS in the training **(A)** and validation **(B)** cohorts based on nomogram-derived risk groups. OS, overall survival.

In the training cohort, the time-dependent ROC curve was drawn and the C-index in the training set was 0.649 (95% *CI*: 0.606-0.692). It showed that AUCs of 3-, 5-, and 8-year were 0.679, 0.683, and 0.708, respectively (**Figure 5A**). These outcomes highlighted the advantageous discriminative ability. Additionally, a calibration curve (**Figure 6A**) and DCA curves (**Figures 7A–C**) were created, affirming that the nomogram demonstrated good calibration and clinical utility. According to the nomogram, patients were categorized into low-risk group (n=301) and high-risk group (n=302). The Kaplan-Meier curve was plotted, indicating that the median OS was 82.6 months for the high-risk group, while it was not reached in the low-risk group (**Figure 8A**). The cumulative OS rates for 3-, 5-, and 8-year were 83.7%, 64.3%, and 45.0% in the high-risk group, while 94.1%, 84.5%, and 73.2% in the low-risk group. There existed an obvious distinction in OS among the two groups (*P* < 0.0001).

### 3.5 Validation of the nomogram

In order to further validate the reliability of this nomogram, we performed internal validation in our study. The C-index in the validation cohort was 0.655 (95% *CI*: 0.542-0.676) and the AUCs for 3-, 5-, and 8-year were 0.701, 0.663, and 0.633, which suggested the favorable diagnostic value (**Figure 5B**). The calibration curve exhibited a good match (**Figure 6B**), and the DCA curves also had good clinical practicability (**Figures 7D–F**). Patients in the validation cohort were then also classified into two groups in light of the nomogram: low-risk group (n=129) and high-risk group (n=130) (**Figure 8B**). The median OS were not reached in both of the low-risk group and the high-risk group. The cumulative OS rates for 3-, 5-, and 8-year were 79.8%, 68.6%, and 50.6% in the high-risk group, while 93.5%, 84.5%, and 74.7% in the low-risk group. In concordance with the training cohort, there was also a statistically significant discrepancy in OS among the two groups (*P* = 0.00058).

## 4 Discussion

HCC remains a challenging malignancy with heterogeneous outcomes, particularly after local ablation therapy (13). In this study, we developed and validated a novel prognostic model integrating WATS to predict OS in HCC patients undergoing ablation. The nomogram demonstrated effective discriminative ability, providing a more individualized risk assessment tool for clinical decision-making.

Local ablation, encompassing techniques such as radiofrequency ablation (RFA), microwave ablation (MRA), irreversible electroporation (IRE), cryoablation, and phototherapy, holds a prominent position in the treatment of HCC (14–16). The ablation process involves inserting an ablation needle into tumor tissues under the guidance of imaging techniques, and high-frequency radio waves are applied to damage HCC cells. Several studies have demonstrated that local ablation provides effective therapy for patients with early-stage HCC during long-term clinical practice experience (17–20). Nevertheless, the prognosis after ablation remains unfavorable, and deserves further investigation.

The WATS score incorporates three key components, including tumor size, tumor number, and AFP level. Larger tumor diameter and multifocal lesions are associated with more aggressive tumor biology, aligning with previous studies demonstrating the adverse impact of increased tumor burden on survival (10). In our study, stratification of patients into high-risk and low-risk groups based on the WATS median value yielded a significant difference in OS, demonstrating its robust discriminatory ability. Compared to conventional staging systems such as TNM or BCLC, which rely on categorical and static criteria, WATS leverages continuous variables (ln AFP) and a weighted algorithm to enhance the precision of risk stratification, particularly for patients with early- to intermediate-stage HCC. Meanwhile, elevated AFP levels reflect not only advanced tumor differentiation but also microenvironmental changes that promote angiogenesis and immune evasion (21). By integrating these parameters into a continuous variable, WATS overcomes the arbitrary cutoffs of traditional staging systems and provides a more nuanced assessment of tumor aggressiveness (8, 22, 23). This refined prognostic assessment may help prevent both overtreatment and undertreatment, thereby overcoming key limitations of traditional staging systems.

To optimize variable selection and enhance model interpretability, we employed a machine learning framework combining Lasso-Cox regression and SHAP analysis (24–27). Lasso-Cox effectively identified the most predictive variables while minimizing overfitting, and SHAP values quantified the contribution of each feature. These methods addressed the limitations of traditional statistical methods by handling multicollinearity and providing transparent, patient-specific risk explanations. The robustness of our model was further supported by its consistent performance across training and validation cohorts, highlighting the potential of machine learning in improving prognostic accuracy for HCC patients who received local ablation. Beyond tumor-related factors, our model identified palb, age, and the history of drinking as independent predictors of OS. Palb, a marker of nutritional status and liver synthetic function, underscores the importance of hepatic reserve in determining post-ablation outcomes (28–30). Advanced age likely reflects diminished physiological resilience and reduced tolerance to salvage therapies, while chronic alcohol use exacerbates liver dysfunction and inflammation, accelerating HCC progression (31–33). These findings emphasize the multifactorial nature of HCC prognosis and the need to consider both tumor-specific and patient-specific variables in clinical decision-making. The inclusion of these factors in our nomogram enhances its predictive accuracy and relevance to real-world patient populations.

The WATS score demonstrates broad potential for clinical translation. It can guide personalized follow-up strategies, with high-risk patients benefiting from intensified monitoring, while low-risk patients undergo less frequent follow-up, reducing healthcare burden and patient anxiety. In the absence of standardized guidelines for postoperative adjuvant therapy, WATS helps identify high-risk individuals who may benefit from targeted or immunotherapies, informing both clinical trial enrollment and real-world treatment decisions. When integrated with a nomogram, WATS facilitates shared decision-making by enabling visual, individualized predictions of survival probability (e.g., 3-, 5-, or 8-year OS), enhancing patient understanding and treatment adherence. Additionally, its reliance on routinely available preoperative clinical parameters (tumor number, tumor size, and AFP) makes it simple to calculate and highly accessible, particularly suitable for resource-limited settings. Furthermore, WATS can serve as a stratification variable in clinical trials and aid in developing more sophisticated predictive tools by integrating emerging biomarkers such as radiomics, liquid biopsy, or immune profiling.

This study has several limitations. First, its single-center, retrospective design may introduce selection bias and information bias, and the characteristics of the study population may limit the generalizability of the findings. Second, although an internal validation cohort was used, the lack of external validation in multicenter, prospective settings restricts the broader clinical applicability of the model. Additionally, there was an imbalance in the distribution of diabetes history between the training and validation cohorts. While this variable was not selected as a significant predictor in the final model (via Lasso-Cox and SHAP analyses), it remains a potential confounding factor, and future studies should ensure balanced representation of comorbid conditions to enhance model robustness.

Future research should focus on validating the WATS score and the associated nomogram in multicenter, prospective cohorts across diverse populations—including varying etiologies, ethnic backgrounds, and treatment modalities. Furthermore, integrating emerging biomarkers—such as circulating tumor DNA (ctDNA), immune microenvironment signatures, or radiomic features—into the existing framework may further improve predictive accuracy. Ultimately, interventional studies are warranted to evaluate whether risk-stratified, WATS-guided management strategies—such as personalized surveillance intervals or adjuvant therapy allocation—can improve long-term patient outcomes.

## 5 Conclusion

Our research successfully created and confirmed a new prediction tool combining the WATS measurement to determine OS in HCC patients treated with local ablation. This comprehensive model effectively distinguished between different risk levels, generating reliable 3-year, 5-year and 8-year OS predictions that can guide doctors in making tailored treatment choices for individual patients.

## Data Availability

The raw data supporting the conclusions of this article will be made available by the authors, without undue reservation.
